# Visual hallucinations after resection of cerebral metastases: two patients with complex phantom images

**DOI:** 10.1007/s00066-024-02213-x

**Published:** 2024-03-07

**Authors:** A. Ovchinnikov, L. Andereggen, S. Rogers, M. Gschwind

**Affiliations:** 1 Department of Neurology, Cantonal Hospital Aarau, Aarau, Switzerland; 2 Department of Internal Medicine, Cantonal Hospital Baden, Baden, Switzerland; 3 Department of Neurosurgery, Cantonal Hospital Aarau, Aarau, Switzerland; 4 Faculty of Medicine, University of Bern, Bern, Switzerland; 5 Centre for Radiation Oncology KSA-KSB, Cantonal Hospital Aarau, Aarau, Switzerland; 6 Department for Clinical Neuroscience, University of Geneva, Geneva, Switzerland

**Keywords:** Brain tumor, Neurosurgery, Neurology, Charles Bonnet Syndrome

## Abstract

**Purpose:**

Complex visual hallucinations are rarely seen in neurooncology. They are commonly observed alongside psychotic symptoms in schizophrenia or dementia, in Parkinson’s or Lewy-body disease, after opioid medications or anesthesia, and, in particular, they appear with visual impairments.

**Methods:**

Here we report two normal-sighted and mentally healthy patients with unusual visual hallucinations after the resection and irradiation of brain metastases, the main features of which were persistent colorful and meaningful images with hallucinatory perseveration.

**Results:**

These cases demonstrate the occurrence of complex visual hallucinations after resection of visual cortices as an effect of deafferentation, so-called visual release hallucinations or phantom images, similar to phantom pain after amputation of a limb.

**Conclusion:**

This case serves to heighten awareness in the radiooncology practitioner of the occurrence of visual release hallucinations (Charles Bonnet syndrome) related to multidisciplinary treatment of brain metastases.

## Introduction

Complex visual hallucinations are a rare symptom of brain metastases which commonly present with visual field deficits such as quadrantanopia or hemianopia. Complex hallucinations differ from simple hallucinations such as dots, lines, phosphenes, or photisms and geometric patterns or colors, which are typically experienced, for example, in migraine aura. Complex hallucinations appear as whole “images” or “scenes,” with people, objects, and animals in defined places, mainly reproduced from the self-witnessed parts of life. Whereas simple hallucinations originate from the retina or primary visual areas, the appearance of complex hallucinations is associated with an imbalance in the visual association cortices and other distributed higher cortical areas [1].

The first studies of damage to the optic pathways resulting in hallucinatory visual symptoms date from the late 1870s, when Hitzig, Ferrier, and Henschen reported on patients with visual hallucinations which, at autopsy, were attributable to lesions in the occipital lobe cortex [2–4]. In 1921, Cushing described 13 cases of visual hallucinations in patients with tumors of the temporal lobe, and Tarachow reported that 96/458 (21%) of patients with supratentorial tumors experienced some form of hallucinations [5]. Colorful, vivid images during wakefulness, mainly at night, without structural changes of the brain tissue have long been a focus of neurological interest. The pathological mechanisms underlying the hallucinations may vary and modern diagnostic techniques enable differentiation between structural, neuroelectrophysiological, and neuropsychiatric etiologies of these symptoms [6–9].

The aim of our report is to provide recent clinical data from two patients who, after resection of occipital and parietal lobe metastases, manifested complex hallucinations without sustained epileptic activity or history of psychiatric diseases, to heighten awareness of this unusual clinical presentation related to the multidisciplinary treatment of brain metastases.

## Case 1

**Fig. 1:**
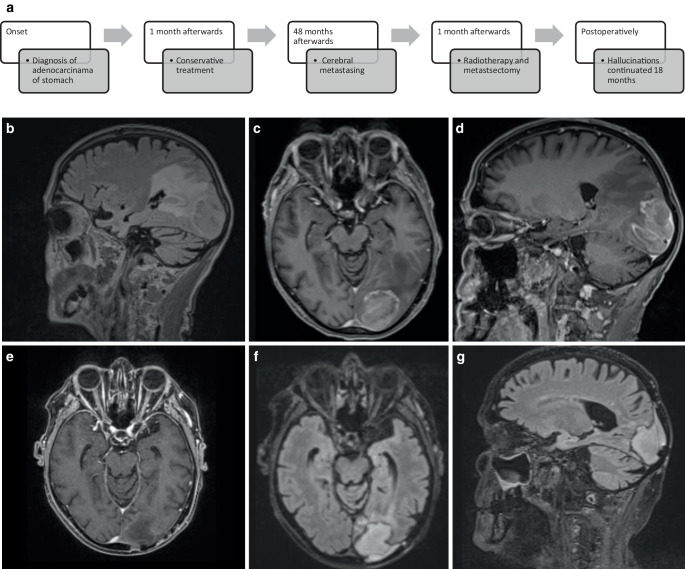
**a** Timeline case 1. **b**, **c**, **d** MRI images at time of diagnosis. **b** Sagittal T2 fluid-attenuated inversion recovery (FLAIR)-weighted sequence depicts a left occipital paramedian dural iso- to hypointense lesion. Axial (**c**) and sagittal (**d**) T1-weighted contrast-enhanced sequences show a slightly hypointense, ring-enhancing, and partially cystic mass with marked perilesional edema. There was a minor midline shift. **e**, **f**, **g** MRI images at 7 months after metastasis resection with subsequent postoperative changes. T1-weighted contrast-enhanced axial image (**e**), T2 FLAIR-weighted axial image (**f**), and sagittal image (**g**) show the resection cavity, with slight enhancement at the margins, and the adjacent sub-/epidural collection in the T2-weighted sequence

A 74-year-old patient presented to his oncologist with binocular hemianopia and persistent left-temporal headache. Four years previously, the patient had been diagnosed with adenocarcinoma of the cardia, which was treated conservatively with paclitaxel and ramucirumab systemic therapies. Clinical neurological examination revealed an incomplete right-sided hemianopia and pre-existing hypoesthesia of the lower extremities, most likely due to paclitaxel-induced polyneuropathy. Cranial magnetic resonance imaging (MRI) revealed a 4-cm paramedian space-occupying lesion in the left occipital lobe. On T2-weighted sequences, the lesion was iso- to hypointense. On T1-weighted images, a hypointense, partially cystic mass with marginal contrast enhancement and pronounced perilesional oedema extending into perisplenic, temporal, and parietal regions up to 4 cm distant to the lesion was visible (Fig. 1b,c,d). The radiological findings were interpreted as an occipital lobe metastasis and a possible cause of the abovementioned visual deficits. The multidisciplinary team recommended neurosurgery followed by radiotherapy, and neurosurgical resection was performed without any surgical complications. The histology confirmed an adenocarcinoma metastasis. We noted a supramarginal resection with acceptance of the tumor–brain interface at the supralateral border facing the visual pathways, resulting in a gross total resection of the metastasis. Postoperative hypofractionated stereotactic radiotherapy with 5 × 5 Gy = 25 Gy prescribed to the 67% isodose was applied to the surgical cavity with a 2-mm margin.

After surgery, the patient mentioned repetitive hallucinations predominantly in the right optic field. They occurred daily, lasting for a few seconds, with no change in the level of consciousness or other epilepsy-suspicious events. The patient recurrently viewed visual images from his past, such as seeing his father, grandfather, or his dog standing in the room, in the right field of vision only. He also reported additional perseveration of a previously seen image (palinopsia) and deformation of central vision (metamorphopsia). The visual field test confirmed a right-sided hemianopia. Initially, an epileptic seizure was presumed to underlie the hallucinations, and antiepileptic therapy with levetiracetam was started. However, an electroencephalography (EEG) performed at that time did not reveal any epileptogenic activity, and a diagnosis of structural epilepsy was considered improbable. The anticonvulsive therapy was gradually discontinued, with no effect on the recurrent hallucinations.

Three months after neurosurgery, the patient reported a slight reduction in the intensity of the daily, brief hallucinations. The previous findings of right-sided hemianopia (predominantly in the upper quadrant with visual neglect to the right) persisted. During the examination, the patient reported a brief hallucination in the right visual field (he saw his grandfather for several seconds) and no additional symptoms could be observed, especially no qualitative or quantitative disturbance of consciousness. The latest EEG without antiepileptic treatment showed no evidence of epileptogenic activity with otherwise regional slowing in the parieto-occipital left region in the context of the resected tumor. Repeated MRI 4 months postoperatively showed no signs of tumor recurrence (Fig. 1e,f,g) and at the latest clinical follow-up 18 months after metastasectomy, the patient did not report any recent hallucinations.

## Case 2

**Fig. 2:**
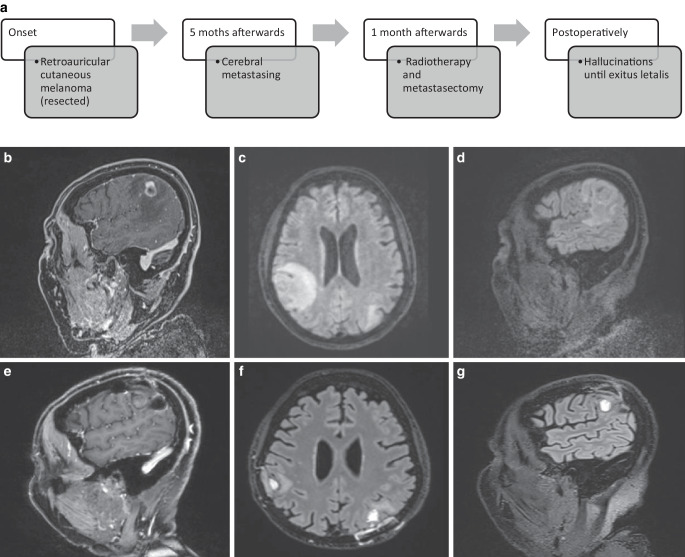
**a** Timeline case 2. **b**, **c**, **d** MRI scans at time of diagnosis. **b** The sagittal T1-weighted image shows an isointense lesion with a ring-like enhancement and perifocal edema. The fluid-attenuated inversion recovery (FLAIR)-weighted image in the axial plane (**c**) and sagittal plane (**b**) depicts a heterogeneously hyperintense space-occupying lesion in the right parietal lobe, surrounded by marked perifocal edema, and also a heterogeneously hyperintense lesion with minor perifocal edema in the inferior parietal gyrus on the left (**c**). There was no midline shift. **e**, **f**, **g** MRI scans 2 months after definitive radiosurgery of the right frontal and left parietal metastases and preoperative radiosurgery of the right parietal brain metastasis followed by osteoplastic craniotomy and resection, and 1 month after salvage left parietal metastasectomy. The sagittal T1-weighted contrast-enhanced (**e**) and T2-weighted fluid-attenuated inversion recovery (FLAIR) sequences axial (**f**) and sagittal images (**g**) show postoperative cavities with regression of the edema

A 76-year-old female suddenly developed weakness in her left arm and leg and slurred speech, with a fluctuating level of consciousness. Computer tomography demonstrated three cerebral space-occupying lesions (right frontal, right parietal, and left parietal) with perifocal edema, presumed to be metastases. Five months previously, the patient had been diagnosed with a retroauricular cutaneous melanoma, which had been fully resected. A subsequent MRI confirmed the two lesions with perifocal oedema (Fig. 2b,c,d), as well as a lesion of unknown origin in the right parietal area. Following multidisciplinary evaluation, right parietal metastasectomy was recommended. Preoperatively, single-fraction radiosurgery was delivered to the right parietal lesion with 1 × 19 Gy. A primary radiosurgery was performed to the right frontal and left parietal metastases (both with 1 × 20 Gy). Almost 70% isodose was delivered to these brain metastases with a 1 mm margin.

Following neurosurgery, the patient was discharged home without complications and the histology confirmed metastatic melanoma. One month later, the patient was re-referred with new neurological deficits of fluctuating global aphasia and confusion. An MRI showed intralesional hemorrhage of the irradiated left parietal metastasis (Fig. 2e,f,g), and 2 days after admission, a navigation- and ultrasound-assisted left occipital craniotomy and tumor resection was performed. As for the postcentral parietal lesion on the right side, also for the parietal lesion on the left side, a gross total resection without supramarginal resection was achieved, with en-bloc resection of the tumor rather than piecemeal tumor extirpation. The tumor bed was inspected, with no residual abnormality noted in either metastasis.

One day after the surgical resection, the patient complained of complex optical visual hallucinations. She stated that she saw a work colleague next to her, with whom she had worked decades previously. She also saw a pregnant woman sitting next to her bed. Once a day, she observed her late husband walk around her room. The hallucinations appeared in all visual fields (both right- and left-sided) and seemed to be restricted to real persons who were not present at the time. In the following weeks, the disease unfortunately led to a fatal outcome.

## Discussion

These two patients presented with complex visual hallucinations after surgical resection of cerebral metastases located in the occipital lobe (case 1) and parietal lobe (case 2), without any relevant neurological, ophthalmological, or psychiatric disorders in the past medical history, and under no opioid pain medication or steroid treatment during the clinical manifestation of the hallucinations.

In the work-up of hallucinations, other competing etiologies of the symptomatology have to be eliminated [10]. From a neurological point of view, it was important to exclude secondary structural epileptic activity of the affected regions, which could also present as polymorphic visual manifestations [11]. However, the multiple EEGs showed no ictal activity in our patients. Paradoxically, antiepileptic drugs such as levetiracetam sometimes potentiate behavioral problems, including visual hallucinations, particularly when combined with steroids [12]. On the other hand, anticonvulsive medication, as used during surgical management of brain tumors, protects against cortical spreading depression (CSD), which also clinically manifests as visual phenomena including photophobia or scintillating scotoma [13]. Only patient 2 had received steroids before the resection, which were then discontinued. High-dose steroids can provoke severe psychiatric complications like delirium, mania, and hallucinations in between 5% [14] to 60% [15] of patients. However, under corticosteroids, hallucinations are part of a psychiatric tableau and do not present alone. Neither of our patients suffered from schizoaffective disorders, neither did they suffer from dementia, which are both also possible etiologies of complex hallucinations [16]. A sudden cortical lesion in the primary visual areas may lead to an unremarked “cortical blindness” or Anton–Babinski syndrome, when the bilateral affection stays filled with clinically manifest hallucinations [17]. In visually impaired individuals, complex hallucinations occur in only 1% of patients, for example as “phantom eye syndrome” [18] or Charles Bonnet syndrome.

More than 250 years ago, the Swiss biologist Charles Bonnet described the hallucinatory experiences of his grandfather, who was suffering from bilateral cataracts [9, 19]. His name has since been linked to the occurrence of visual hallucinations predominantly in elderly individuals with normal mental health and cognitive performance, but with a degree of visual loss as a consequence of a disease of the visual system [8]. This type of complex visual hallucination has been conceptualized as “phantom vision” due to deafferentation, similar to phantom-limb syndrome [8].

Regarding the localization of the metastatic lesions in our patients, postoperative sites in the occipital and parietal regions do not necessarily lead to deafferentation of the basic visual pathways such as in case 1, but can also affect the higher visual association cortices, such as in case 2. Visual inputs are assumed to be regulated by the inhibition processes of higher cortical areas, and damage to these cortical structures leads to impaired suppression of neuronal excitation, which is followed by biochemical changes and an increase in excitability with a releasing of the visual symptoms [10]. Some authors describe that brainstem lesions may also lead to impairment of the ascending cholinergic and serotonergic pathways, which can be manifest with complex visual phenomenon (called “peduncular hallucinosis”) [7].

Phantom vision due to deafferentation is a real symptom, explained by a phenomenologic and neuroscientific concept. However, it is formally impossible to decide the exact pathomechanism at the neuronal level between interruption, micro-epileptic activity, or CSD within the postsurgical lesions, but there is also no need to do so. There is currently no evidence for any treatment of Charles Bonnet syndrome, in particular after surgical tumor resection. According to the literature, the pharmacological targets in the management of the hallucinations modulate predominantly GABAergic, dopaminergic, cholinergic pathways or even apply a reduction of the cortical excitation. Antipsychotic drugs are frequently used to alleviate the visual hallucinations in Parkinson’s disease and delirium [20]; whether they are also useful in patients with structural cerebral lesions remains open. Some authors describe a beneficial use of antiseizure drugs to reduce the frequency and intensity of the symptoms in similar cases [7, 21], but this reduction of the cortical excitation with levetiracetam did not diminish the symptoms in our patients; thus, it also sustains our hypothesis of a nonepileptic origin of the hallucinations. Generally, previous studies concluded that there is no need for pharmacologic treatment unless the patient is particularly bothered and intimidated by the hallucinations. Most important is that the healthcare provider recognizes the syndrome to avoid misdiagnosing mentally healthy individuals, which can lead to unnecessary and potentially harmful treatment. The duration of Charles Bonnet syndrome is in most cases self-limited, and the information about the benign course is also reassuring for the patient [7–9, 20, 21].

## Conclusion

Patients with space-occupying lesions in the occipital lobes often present with visual symptoms. Whereas visual field defects are well-recognized symptoms that suggest cortical or subcortical occipital lobe damage, the cases presented here highlight that also complex visual hallucinations resembling psychiatric symptoms can be a clinical manifestation of organic cerebral lesions. Being familiar with the symptoms of visual release hallucinations of Charles Bonnet syndrome helps the radiooncologist to correctly recognize this phenomenon and avoid misdiagnosing mentally healthy individuals. Charles Bonnet syndrome is in most cases self-limited, and no treatment is needed.

## Abbreviations

CSD: Cortical spreading depression
EEG: Electroencephalography
FSRT: Fractionated stereotactic radiotherapy
MRI: Magnetic resonance imaging
SRS: Stereotactic radiosurgery
